# Collagen Mimetic Peptides

**DOI:** 10.3390/bioengineering8010005

**Published:** 2021-01-05

**Authors:** Yujia Xu, Michele Kirchner

**Affiliations:** Department of Chemistry, Hunter College of the City University of New York, 695 Park Ave., New York, NY 10065, USA; mkirchne@hunter.cuny.edu

**Keywords:** collagen mimetic peptides, fibril-forming collagen peptide, homotrimer triple helix, heterotrimeric triple helix, recombinant collagen peptides, design of collagen mimetic peptides, collagen receptors, collagen-based biomaterials, extracellular matrix, synthetic collagen

## Abstract

Since their first synthesis in the late 1960s, collagen mimetic peptides (CMPs) have been used as a molecular tool to study collagen, and as an approach to develop novel collagen mimetic biomaterials. Collagen, a major extracellular matrix (ECM) protein, plays vital roles in many physiological and pathogenic processes. Applications of CMPs have advanced our understanding of the structure and molecular properties of a collagen triple helix—the building block of collagen—and the interactions of collagen with important molecular ligands. The accumulating knowledge is also paving the way for developing novel CMPs for biomedical applications. Indeed, for the past 50 years, CMP research has been a fast-growing, far-reaching interdisciplinary field. The major development and achievement of CMPs were documented in a few detailed reviews around 2010. Here, we provided a brief overview of what we have learned about CMPs—their potential and their limitations. We focused on more recent developments in producing heterotrimeric CMPs, and CMPs that can form collagen-like higher order molecular assemblies. We also expanded the traditional view of CMPs to include larger designed peptides produced using recombinant systems. Studies using recombinant peptides have provided new insights on collagens and promoted progress in the development of collagen mimetic fibrillar self-assemblies.

## 1. Introduction

The term collagen *mimetic* often conjures up two different ideas: Those that intend to capture the biological functions of collagen by mimicking the structural hierarchy of collagen building up from the triple helix, and those “inspired” by the properties of collagen and trying to mimic its nano-scale structure and function using non-biological polymers. Examples of the latter include the molecular scaffold made of electrospun polymers with a similar diameter and morphology as collagen fibrils, or nano-scale tubes self-assembled from non-peptide building blocks but decorated with certain amino acid residues on the surface mimicking the functions of collagen [1,2,3,4,5]. Applications of collagen mimetic peptides (CMPs) belong to the former. Peptides are developed to resemble collagens in their amino acid sequence, in their structure, and in their bioactivity. The principle of such an approach falls within the general premise of structural biology that at the foundation of the biological functions of a biomolecule is its molecular structure.

### 1.1. The Macromolecular Assembly of Collagen

Collagen is a family of extracellular matrix proteins with considerable diversity both in structure and in function. A total of 28 different types of collagen have been identified in this super family, among which the fibrillar collagens are the most abundant and are also the best characterized [6,7,8]. The major fibrillar collagens include collagen types I, II, and III. Collagen type I is the major collagen in bones, skin, and tendon. Collagen type II presents primarily in cartilage. Type III collagen often coexists with type I in skin, and in blood vessel walls. Other types of fibrillar collagens are present at a lower amount and are often found coexisting with the three major types.

**Figure 1:**
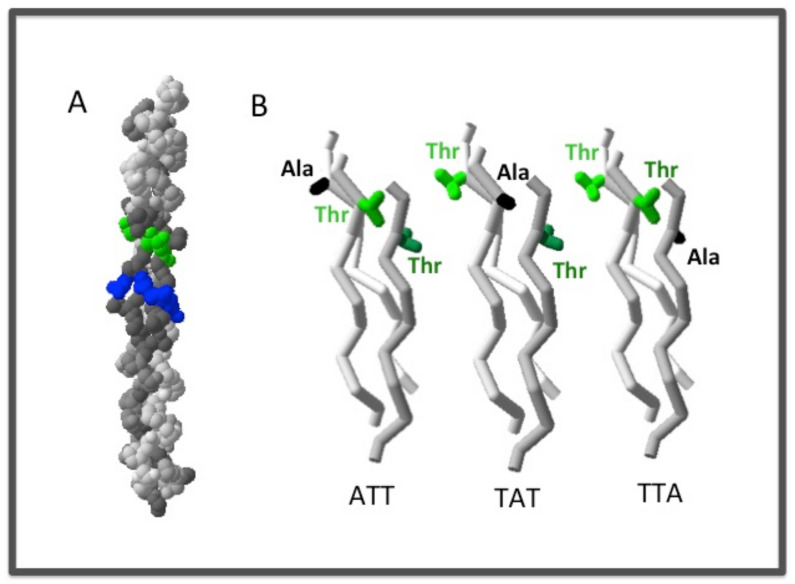
The rod-shaped conformation of the triple helix. (**A**) The structure of the homotrimer triple helix T73–785 [15] was generated using DeepView–Swiss-PdbViewer (PDB: 1bkv). The helix is shown with the N-terminus on top; the Thr residues are shown in green, Arg in blue, hydrophobic residues in dark gray. (**B**) In order to show the asymmetric structure of a heterotrimer associated with different chain registers, a type I collagen like the AAB heterotrimer model was created using DeepView by replacing the Thr of one of the three chains of 1bkv to Ala (side chain shown in black). The amino acid residues included in this heterotrimer model are ITGARGLAG for the two identical strands, and IAGARGLAG for the third, mutated strand. The three structures are shown in the identical view of the backbone, with the N-terminus on top.

The structural hierarchy of all collagens starts from the building block: The collagen triple helix [8,9]. A collagen triple helix consists of three polypeptide chains (often referred to as the α chains) coming together in parallel with a precise one residue staggering at the ends [9,10]. The three chains tightly wrap around each other about a common axis in a right-handed helical twist to form a rod-like helical conformation (Figure 1A). The tight packing of the triple helix requires a Gly residue at every third position, giving rise to the characteristic (Gly-X-Y)_n_ repeating sequence. The obligatory Gly residues are buried at the center of the helix, the side chains of X and Y residues are largely exposed to solvent. The triple helix is often considered a “linear molecule” because of its uniform backbone conformation characterized by an ~0.86 nm helical rise per Gly-X-Y tripeptide [11,12,13,14]. The side chains of the X and Y residues can be described as a linear sequential array in an N-to-C directionality spiraling around the surface of the molecule (Figure 1A).

The three polypeptide chains of a triple helix can be identical in the form of a homotrimer, or they can be different in amino acid sequences forming a heterotrimeric triple helix [8]. Collagen type II and collagen type III are homotrimers, while collagen type I is a heterotrimer consisting of two identical α1 chains, and one α2 chain. There is about 72% sequence similarity between the two α chains in the triple helix domain of type I collagen. Because of the one residue stagger between the adjacent strands in the triple helix, the analogous residues in each strand are unique even in a homotrimer environment (Figure 1A) [10,16,17,18]. The three strands are usually called leading, middle, and trailing, as viewed from their N-termini. The chain-stagger-related asymmetry in structure is particularly pronounced in a heterotrimer (Figure 1B). Thus, for type I collagen the surface features of the triple helix can be very different depending on which chain is in the leading, middle, or trailing position. There are three possible chain registers for type I collagen: α1α1α2, α1α2α1, and α2α1α1, which are often referred to as α2 trailing, α2 middle, and α2 leading, respectively. Unfortunately, determining the chain register is not at all easy. The correct chain register of type I was accepted to be α1α2α1 [19,20]. Emerging data from studies using CMPs, however, are challenging this chain alignment in favor of an α1α1α2 register with the α2 chain in the trailing position (details below) [21]. Inside the cell, the C-terminal globular domain, the C-propeptide, was believed to be responsible for both chain selection and chain registration [22]. Structural studies of type I collagen C-propeptide have provided a mechanism for heterotrimerization of the C-propeptide. How the structure of the C-propeptide determines the chain alignment of the triple helix domain, however, remains a mystery [23,24].

**Figure 2:**
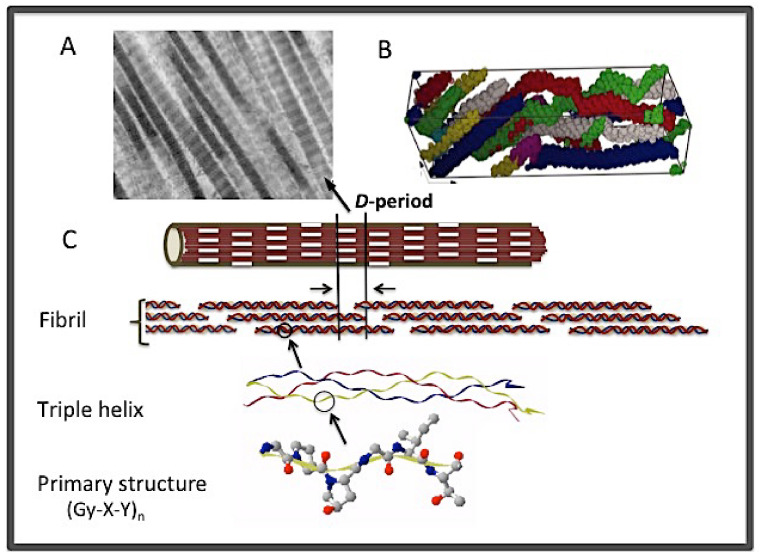
The structural hierarchy of fibrillar collagen. (**A**) Electron micrograph of collagen fibrils showing the characteristic striation pattern of the *D*-period and the tipped ends. (**B**) The unit cell of collagen fibril showing the staggered and intertwined arrangement of five triple helices (in different colors) due to the super-twist of the triple helices in fibrils [33]. (**C**) The different stages of fibrillogenesis from the primary structure to fibrils.

Collagens in tissues are higher order, supramolecular assemblies of triple helices. Fibrillar collagens self-associate laterally with a specific 67 nm staggering at the ends to form fibrils (Figure 2A,C) [25,26,27,28,29,30]. Fibrillar collagens are large molecules consisting of more than 1000 residues per single polypeptide chain in uninterrupted (Gly-X-Y) repeating sequences, forming a long triple helix about 300 nm long and ~1.5 nm in diameter. Each triple helix compromises about 4.4 × 67 nm in its total length. The staggered arrangement would thus generate long, smooth fibrils with alternating gap-and-overlap regions every 67 nm. This 67-nm structure is termed a *D*-period, which consists of a 0.4*D* overlap zone and a 0.6*D* gap region. The overlap and the gap zones appear as light and dark bands, respectively, when examined using an electron microscope, giving rise to the characteristic striation appearance of collagen fibrils (Figure 2A). In the fibrils, the triple helix further adopts a right-handed super-twist around the microfibrils [31,32,33,34,35]. Because of this super-twist, there is an uneven exposure of different parts of the triple helix on the fibril surface (Figure 2B); as to which specific sections of the triple helix might be exposed on the surface of the fibrils is still under debate [32,36,37].

The *D*-period of collagen fibrils is an important feature that has been linked to the tensile strength of bones, the stiffness of the extracellular matrix, and other biomechanical properties of tissues [38,39,40,41,42,43]. It is the holy grail of all development of collagen mimetic biomaterials to capture the structure and function of this unique, yet ubiquitous molecular scaffold of all connective tissues. Some of the recent progress in achieving collagen mimetic fibrils is mentioned later in this review. Fibrillogenesis in tissues is a complex process and remains poorly understood. The fibril assembly process per se, however, is a self-assembly process that can be reproduced in vitro from acid-dissolved fibrils [44,45]. It is generally considered that the axial repeating *D*-period of fibril assembly is determined by the molecular interactions contained in each triple helix, although the exact molecular mechanism is not resolved [11,12,13]. Alternatively, some studies have attributed the deterministic factor of fibrillogenesis to the involvement of the telopeptides: Two short stretches of peptides at the N- and C-termini of the triple helix domain that do not confer to the Gly-X-Y sequence pattern and do not adopt to a triple helix conformation [46]. Later studies have shown that triple helices without the telopeptides can form fibrils, albeit with a slower kinetics [47,48,49].

Collagen plays much more than a structural role in tissues. It is a dynamic molecular scaffold that supports cell adhesion, cell migration, and cell differentiation [36,50,51,52,53,54]. Cell receptors recognize specific regions of the triple helix. Studies using CMPs to identify these recognition sites are described in Section 2 below, and a comprehensive review of the distribution and organization of these epitopes is given by San Antonio et al. in this special issue. One major family of collagen-binding proteins is integrin. Integrins are the major cell adhesion proteins that bind to the extracellular matrix (ECM) and function as signal transducers of various signaling pathways that can induce global cell responses and affect gene expression [55]. Epithelial and endothelial cells will undergo programmed cell death, or apoptosis, when they lose contact with the ECM. Cells can also control their affinity to collagen through inside-out signaling. The intricate interplay between cells and the ECM is integral to the development of all tissues and organs. Other important collagen receptors include discoidin domain receptor (DDR1 and DDR2), platelet glycoprotein GPVI, immune receptors, the plasma protein von Willebrand factor (vWF), and other macromolecules and proteins [21,36,51,56].

Collagen catabolism is another critical interaction of collagen for maintaining the ECM homeostasis [57]. The hydrolysis of fibrillar collagens is due to the action of matrix metalloproteinases (MMPs) MMP-1, MMP-8, and MMP13. All three enzymes cut collagens into ¾ and ¼ length fragments, but with different kinetics to different collagens. MMP-8 preferentially cleaves type I collagen, while MMP-1 has greater catalytic activity on the type III collagen. MMP-13 cleaves type II collagen 5 and 6 times faster than collagens type I and type III, respectively. MMP-2 and MMP-9, which are broadly categorized as gelatinases, also participate in the homeostasis of the ECM by hydrolyzing a partially unfolded, or gelatin form of collagens [58,59]. The well-regulated digestion activity of the MMPs controls the turnover of collagen in normal growth and in tissue remodeling. The unregulated proteolysis by MMP is also the cause of pathological conditions such as arthritis, periodontal diseases, and cancer metastasis [60,61,62].

Another type of collagen which has often been studied using CMPs is the basement membrane collagen–collagen type IV. Unlike fibrillar collagen, type IV collagen self-assembles into a chicken wire-like molecular network joined at the ends through the N- and C-globular domains. The molecular composition of type IV collagen is also quite complicated [8]. There are six different α chains of type IV collagen that form at least three distinct triple helices with the stoichiometries of 2α_1_α_2_, α_3_α_4_α_5_, and 2α_5_α_6_, respectively. Different isoforms are found in different tissues, and in different developmental stages. While the molecular assembly of the type IV collagen may not have the biomechanical properties of fibrillar collagen, it is the critical molecular scaffold of the basement membrane supporting angiogenesis, and has many cell recognition sites [63]. Molecular interactions with type IV collagens are involved in cancer metastasis; these interactions are potential targets of cancer drugs [64,65,66].

We are just starting to understand the structural and the molecular details of collagen, and the interactions involving collagen. As is described in more detail, CMPs have been indispensable in these studies. Many reactions take place in an overcrowded ECM environment where collagen triple helices organize into different molecular scaffolds or networks. How the supramolecular structures of collagen modulate the cell-ECM interactions is yet to be fully elucidated.

### 1.2. Collagen-Based Biomaterials

Collagen-based biomaterials in the form of sutures and films for wound healing have been used for more than a century [67]. The field has grown tremendously since then to include a broad range of potential medical products based on collagen. The clear advantage of collagen devices is their ability to interact with the host. These products can act as a scaffold for new tissue formation prior to resorption, or be used for soft tissue augmentation. Collagens from animals are often the source for manufacturing these materials. However, the purification process of collagen from animal tissues is a difficult and expensive process. Because of the constant tissue remodeling and modification of collagen, collagen preparations isolated from tissues are often heterogeneous with a high degree of variations in terms of covalent modifications and composition. While collagens per se are considered poor immunogens, the impurities in collagen preparations are known to elicit immunogenic responses. In recent years, there have been increasing concerns of the pathogenicity of collagen devices made from animal collagens or from collagen of human tissues.

Collagen mimetic peptides, thus, represent a desirable alternative for being safer and potentially less expensive. The one other great advantage of mimetic materials, whether produced by chemical synthesis or by genetic engineering, is the ability to modify the amino acid sequences for a better control of the properties and activities of the material. The goal of the collagen mimetic materials should mimic both the physical and biochemical properties of native collagen. Additionally, control of the turnover rate is another important factor for the proper tissue remodeling process, and also for the life span of the material. CMP-based biomaterials bearing epitopes for platelet activation have already demonstrated their potential for application [68,69]. The effectiveness and value of the collagen mimetic biomaterials will largely depend on the ability to modulate and to fine-tune the bioactivities of the material for specific applications.

## 2. Collagen Mimetic Peptides by Chemical Synthesis

### 2.1. The Homotrimeric CMPs

Synthetic peptides have been an integral part of protein science. The collagen field is no exception. What is unique to collagen is the requirement to have three peptide chains come together and fold into a triple helix conformation with a specific one-residue stagger at the ends. An early approach was to use chemical cross-links at the C-terminus to bring about the correct association of the three polypeptide chains [70,71]. Later, it was found that peptides with repeating sequences of (Gly-Pro-Hyp)_n_ with n > 6 can self-assemble into a stable triple helix without the need of cross-links, and the thermal stability increases with an increase in the number of tripeptide units [72]. Thus, while the triple helix (Gly-Pro-Hyp)_6_ was only marginally stable with a melting temperature (*T_m_*) of 10 °C, that of a (Gly-Pro-Hyp)_10_ can reach 68 °C. Peptides with (Gly-Pro-Pro)_n_ repeating sequences also form a triple helix, but have a much lower thermal stability; in comparison with (Gly-Pro-Hyp)_10_, the thermal stability of (Gly-Pro-Pro)_10_ is only about 27 °C [73,74]. Since these pioneering studies, it has become quite feasible to synthesize CMP 21–50 residues in sizes with more sequencing variety. The only requirement is to follow the (Gly-Xxx-Yyy)_n_ sequence pattern with Gly at every third position, although repeating (Gly-Pro-Hyp)_n_ or (Gly-Pro-Pro)_n_, with n = 2–4, are frequently included at the N- and/or C-termini for added stability [10,75]. Later studies showed that the multiple (Gly-Pro-Hyp)_n_ or (Gly-Pro-Pro)_n_ at the end of the peptide can also function as the nucleation domain to facilitate the association of the three chains in the desired mutual one residue staggering [76,77,78,79]. The spontaneous folding of such CMPs inevitably leads to homotrimeric triple helices consisting of three identical polypeptide chains.

#### 2.1.1. The Sequence–Structure Relationship

Studies using the CMP have clearly demonstrated that the rod-shaped triple helix is not uniform in structure or stability [10,80,81]. The three polypeptide chains of a triple helix are connected by a set of hydrogen bonds between the backbone NH of Gly and the backbone CO (X-position) of the neighboring chain [15,82,83,84]. These main-chain H-bonds present rather uniformly throughout the helix. Additional water-mediated H-bonds can be found between the CO of the Gly with the NH of an X-residue if the X-residue is not a Pro. Crystal structures of CMPs also revealed a sequence-dependent variation in the helical twist. Regions with a high content of Gly-Pro-Hyp or Gly-Pro-Pro form a tighter 7/2 helix (3.5 residues/turn), while the “imino acid-poor” regions adapt to a more relaxed 10/3 symmetry (3.3 residues/turn) [9,10]. This sequence-dependent variation can potentially play a role in the molecular recognition of collagen.

The stability of the CPMs is sensitive to the residues in the X and/or Y positions. Using a set of host–guest peptides, Brodsky and colleagues examined the effects of all 20 amino acid residues plus hydroxyproline in X and/or Y positions(s), and in different combinations [80,81,85,86,87,88]. The Gly-Pro-Hyp was the most stable tripeptide; the host peptide (Gly-Pro-Hyp)_8_ had a *T_m_* of 47.3 °C in neutral buffer. Replacing the Pro in the *guest* site positioned in the middle of the peptides often resulted in a decrease of *T_m_* ranging from 2–15 °C depending on the identity of the substituted residue, while that caused by replacing Hyp in the Y position ranged from 0 to 21 °C. Having a charged residue like Glu, Asp, Arg, or Lys generally had an unfavorable effect with the exception of Arg in the Y position, which appeared to have a similar stabilizing effect as a Hyp. However, when the charged residues were present in pairs in the sequences of Lys-Gly-Glu (KGE) or Lys-Gly-Asp (KGD), significant stabilizing effects were reported: The *T_m_* of a host–guest peptide with a sequence G_KGE_ or G_KGD_ can increase by 15.4 to 17.5 °C, respectively. This significant stabilizing effect was attributed to a set of inter-chain salt bridges between a pair of oppositely charged residues. In the extended backbone conformation of the triple helix, little interactions could take place between the charged groups in the same chain. Furthermore, the effects of different residues on the overall stability of the triple helix appeared to follow a simple additive rule. A stability calculator was developed based on these studies that could provide reasonably accurate estimations of the thermal stability of CMP 18–50 residues in size, and has been used broadly in the sequence design of homotrimeric CMPs [80].

As the peptide becomes longer, the triple helix is more stable until it reaches a plateau at about 50 residues or so. An empirical curve was used to predict the length dependence of the CMPs [80]. For longer chains, the decrease in entropy of the polypeptide chains in the more constrained folded structure could off-balance the enthalpy contribution in the form of H-bond formation and other interactions. This entropy penalty is more significant for the folding of a longer triple helix. A more quantitative interpretation of the length dependence on the thermal stability would require thermodynamic studies under an equilibrium condition. The thermal unfolding process of CMPs generally does not satisfy this condition [9,81]. How to extrapolate the sequence–stability relationship of CMPs to natural collagen also remains an intriguing question, since natural collagens are not only much longer, but also have much more diverse amino acid sequences. This subject will be revisited in Section 3 on the discussion of recombinant peptides. The findings of the CMPs are generally applicable to larger triple helices, at least on the qualitative level.

#### 2.1.2. The Binding Sites of Collagen Receptors

Defining the binding sites of cell receptors and other macromolecules on collagen is considered one of the crown accomplishments of CMPs [21,50]. Given the complex supramolecular structure of collagen in the ECM, defining the site where a collagen-binding ligand may interact is difficult. The collagen-binding proteins themselves are also frequently membrane-bound molecular complexes, or in the case of vWF, a complex multi-domain protein. CMPs carrying 6–27 residues modeling a selected section of the α1 chain of collagen type I were used to study the interaction of collagen with integrin [89,90]. The I-domain (or A-domain) of the α-subunit of integrin α_2_β_1_ and α_1_β_1_ was identified as the domain to interact with both collagen types I and IV in a metal-dependent fashion [91,92,93]. Peptides containing the sequence GFOGER were first selected as potential binding sites because of their high affinity to the isolated I-domain and ability to support α_2_β_1_-mediated cell adhesion. Subsequent studies using mutations indicated that the recognition of the I-domain is entirely contained in the six-residue sequence, with the residues Arg (R) and Glu (E) being the most critical for the binding [90].

The molecular recognition mechanism of collagen binding by integrin was revealed by the crystal structure of the complex of the I-domain with a 27 mer CMP containing GFOGER at the guest site [94]. The Glu from the middle strand of the triple helix formed a critical interaction with the required divalent ion, and the Arg residue from the middle strand formed a salt bridge with Asp219 of the I-domain to stabilize the complex. The fact that both the Glu and Arg involved in specific interactions with the I-domain all came from the middle strand of the triple helix appeared to offer an explanation as to why the homotrimeric peptide binds the I-domain with similar or even higher affinity as that of heterotrimeric collagen type I. The sequence of the alpha 2 chain of type I collagen in the equivalent location is GPOGES. In the molecular complex, the binding site of the I-domain had close contacts with only one out of the three chains; the interactions with the residues of the other two chains may have facilitated the binding with less specific interactions. The Phe in the middle, and the trailing strands made hydrophobic contacts with the I-domain, while the one in the leading strand was exposed on the surface of the complex. The hydrophobic residues Ala and Leu in the other high-affinity sequence GAOGER and GLOGER, respectively, were expected to provide similar interactions during binding [95]. A specific involvement of Hyp in the six-residue binding site was suspected because of its common presence in the identified high affinity sites. The Hyp of the trailing strand was buried in the interface of the complex, a replacement of residues with larger side chains are likely to create steric clashes and decrease the affinity. No other, more specific interactions involving this Hyp were identified. Later studies found replacing the Hyp with Pro reduced binding affinity but did not abolish the integrin activation [96]. Studies using cross-linked heterotrimeric peptides have indicated the binding affinity of the three different chain registers showed similar affinity to the I-domain. Interestingly, the binding affinity of the heterotrimers was significantly lower than that of the homotrimer.

The peptide approach was later developed into a system known as the ToolKit III [50,95,97]. The ToolKit III consists of 57 peptides with overlapping sequences at the 27-residue guest site that cover the entire sequence of type III collagen. Repeating GPO or GPP sequences flanking the guest site were included to facilitate the triple helix formation. Since sequences of (GPO)_n_ can interact with the GPVI of platelet, the (GPP)_n_ sequence is preferred especially for studies with platelets [98]. The ToolKit approach is particularly good when studying the interactions of collagen receptors with collagen type III and collagen type II, due to their homotrimeric nature. Applications using the ToolKit peptides led to the identification of the epitopes of integrin α1β1, α2β1, the vWF on type III collagen, and the DDR2, DDR1, and the immune receptors on type III collagen and type II collagen [97,99,100,101,102,103]. Ideally, these peptides should be covalently crosslinked as well, since the trimerization process of triple helix folding is sensitive to peptide concentration. For this reason, the peptides of the ToolKit III have a Cys at the N- and the C-termini. Given the triple helix conformation, two adjacent Cys residues are often needed in order to cross-link all three chains in a set of disulfide bonds; a single Cys in a peptide can only cross-link one other neighboring chain and leaves the –SH on the third chain unpaired. Peptides with free –SH groups are often prone to non-specific aggregations.

In tissues, these molecular interactions with collagen receptors take place with collagens in fibril form or in other supramolecular structures. Some epitopes showing a high affinity in a CPM study may not be fully exposed on the surface of the packed fibrils (Figure 1B) [32,36,37,104]. Even if the specific molecular interactions are available, the binding kinetics and binding affinity are likely to be affected by the structural context of the epitopes. Molecular modeling indicates that an I-domain should be able to bind to a triple helix without steric clashes with neighboring helices on the fibrils based on the center-to-center distance of the closely packed triple helices being 15 Å in fibrils [94]. However, the I-domain is also just a part of the integrin complex and does not work in isolation. It remains to be tested in studies of the I-domain and other collagen-binding proteins and/or domains with the triple helix in a higher molecular complex.

### 2.2. The Heterotrimeric CMPs

In the past decade or so, progress has been made in designing and creating heterotrimeric collagen peptides. Two of the major collagens, collagen type I and collagen type IV, are heterotrimers, and are known to interact extensively with cell receptors. Heterotrimeric peptides are used as model systems to understand these interactions [19,21,96]. Making heterotrimeric triple helices with the correct register faces many challenges; not the least of them is the lack of clear knowledge of the actual chain registration of the native collagens. Because of the one residue staggered arrangement, a triple helix formed from two or three different peptide chains will create a different structure environment for the interaction with cell receptors (Figure 1B).

#### 2.2.1. The Chain Register Affects both the Stability and the Binding Affinity of the Triple Helix

As it was in the case for the homotrimer CMPs, terminal cross-links were first used to circumvent the problems of the chain selection and chain registration. To gain more detailed characterization of the mechanism of proteolysis of collagen type I by the metalloproteinase, a hetero CMP was synthesized to mimic the MMP-1 digestion site between residues 772–783 of the α1 and α2 chains of type I collagen [19,105]. Altogether, 4 Cys residues were included at the C-terminus of the peptide chains which were designed to cross-link the three chains through two disulfide bonds. The chain register was fixed to α1α2α1 (α2 as the middle chain). An additional (GPO)_5_ at the N-terminus was also included to give the peptide a thermal stability of ~33 °C. The enzyme digest assays performed at room temperature indicated the triple-helical substrates were cleaved with a single cut through the three chains in a manner similar to those observed in studies using collagens isolated from tissues. This similar enzyme activity was also used to argue that the chain register of type I collagen must be α1α2α1. However, an NMR study of the peptide showed the C-terminal half of the peptide appeared to be disordered. It is not clear if the disordered conformation was due to the low helix propensity of the sequences or the structural constraint imposed by the C-terminal cross-link. Later studies by crystallography also indicated that the regions surrounding the interchain disulfide bonds are often flexible and the chain register may not be fixed by their use. Additionally, there is a chance the disulfide bond may reshuffle, and lead to unexpected cross-links [106,107].

Another set of similar cross-linked hetero-CMPs were used to model the α1β1 binding sites of collagen type IV. The study revealed the chain register affected both the stability and the folding kinetics of the triple helix [108,109]. The trimer of the α1α2α1 register was more stable (*T_m_* of 42 °C) but had lower affinity to integrin, while the trimer with a α2α1α1 was less stable (*T_m_* of 30 °C), but showed higher affinity [108,109]. The high stability was postulated to affect the binding because collagen needs to undergo conformational changes in the integrin adhesion region upon binding, and a less rigid conformation may favor such a conformational adjustment.

#### 2.2.2. The Self-Assembled Heterotrimeric Triple Helix

A heterotrimeric triple helix formed by self-association of three polypeptide chains without cross-linking is not just an interesting, fundamental problem to solve for protein design, but one that would offer more flexibility in chemical synthesis and more versatility for applications. The challenge is the control of both the chain composition and the chain register. The control of composition is a tractable problem if different compositions lead to different stability. By simply mixing two peptides A and B, there will be 8 possible combinations of trimers: AAA, BBB, ABB, BAB, BBA, AAB, ABA, and BAA; where the AAB and ABA have the same chain composition but represent two different triple helical structures due to the different chain register. The population of each of the 8 possible configurations is partitioned into a Boltzmann distribution based on their stability; the desired configuration can be the dominant species if its stability is significantly higher than any of the other competing species. The control of the chain register turns out to be a closely related problem (see below), since the chain registers also affect the stability of the triple helix.

One stabilizing factor that can be exploited for the design of a stable heterotrimeric triple helix is the inter-chain salt bridges [80,86,88]. By strategically placing a pair of oppositely charged residues on neighboring chains, the interactions between the charged pair lead to the formation of a salt bridge (an ionic H-bond) that can significantly stabilize the triple helix. In contrast, unpaired charged residues in the X or Y position are known to cause destabilizing effects on the triple helix due to charge repulsion, and can be used to discourage unwanted conformations. Nanda and coworkers implemented this idea and computationally generated three peptide sequences A, B and C, which by mixing in a 1:1:1 ratio formed an ABC heterotrimer (here ABC stands for chain composition not chain registration) [110,111]. By several rounds of optimization to increase the stability of the ABC trimer while destabilizing other competing configurations, the ABC trimer emerged as the only triple helix in the solution. However, the ABC heterotrimer could have 6 possible chain alignments. The most likely alignment was the one designated as *abc* with A, B, C chains in the leading, middle, and trailing positions, respectively. Based on the computer simulation, *abc* had the most favorable charge-paired interactions and fewest charge repulsions and thus, should have been the one that had the highest stability. Later, a crystal structure study of the designed ABC heterotrimer confirmed the *abc* chain alignment [112]. The amino acid sequences of the A, B, and C peptides, however, were nothing close to that of natural collagen. It will be interesting to find out if some of the sequence features can be utilized to generate heterotrimers with more sequence diversity.

Using an intuitive experimental approach, Hartgerink and coworkers exploited the geometric and sequence specificity of electrostatic interactions of charged residues nearly exhaustively in a series of 30-residue peptides [113,114,115,116,117,118,119,120,121]. Among the major findings are that peptides carrying 5 or more like charges such as (DOG)_10_ or (PKG)_10_ will not form homotrimers because of a strong charge repulsion. However, when such decapositive and decanegative peptides are mixed with (POG)_10_ in a 1:1:1 molar ratio, they form stable ABC heterotrimers. Most of all, the NMR study shows that such heterotrimers are stabilized by a set of axially oriented charge–pair interactions between a Lys residue in the leading and middle chains with a Glu or Asp residue in the middle and trailing chains, respectively; with the interactions between the Lys-Asp being particularly strong. Furthermore, the orientation bias of the Lys-Asp interaction means only in the alignment of (PKG)_10_(EOG)_10_(POG)_10_ (i.e., with (PKG)_10_, (EOG)_10_, and (POG)_10_ in the leading, middle, and trailing positions, respectively) can all 10 sets of salt bridges be satisfied, and this maximized stability predetermined that only heterotrimer in this chain register will dominate in the solution. The same type of the axial Lys-Asp salt bridge was also found in the crystal structure of the heterotrimer mentioned above by Zheng et al. [112]. This directional interchain charge–pair interaction has since been used to develop self-assembled heterotrimers or a long super-helix [21,122,123].

Another equally important consideration in designing a stable heterotrimer is to destabilize the competing compositions. While the original ABC heterotrimer based on the inter-chain charge–charge interaction was remarkably strong, with a *T_m_* above 56 °C, they cannot outcompete the homotrimer (POG)_10_, which has a *T_m_* of 58 °C [115]. Thus, mixing of the three peptides generated a mixture of heterotrimer and the (POG)_10_ homotrimer. In another effort, Hartgerink and coworkers developed a stable ABC heterotrimer using a decapositive (PKG)_10_ and a decanegative (EPG)_10_ mixed with a zwitterionic peptide (DKG)_10_. Since the zwitterionic peptide does not form a stable homotrimer, the ternary mixture produced a single component ABC heterotrimer with a single chain registration (117). The peptide has a *T_m_* of 38 °C which is quite remarkable considering this trimer has no Hyp.

By maximizing the axial Lys-Asp interactions while reducing the possibility of like-charge repulsions at the same time, a stable heterotrimer in the AAB arrangement was achieved in the composition of 2(GPKGEO)_5_(POGDOG)_5_ [119,120]. The heterotrimer has a unique AAB chain register because only in this chain alignment are the interactions of the directional, axial Lys-Asp salt bridge maximized, while unfavorable, unpaired charge residues are minimized. This study is a clear demonstration that, by strategically placing the charged residues, both the chain composition and the chain register can be controlled. At the same time, however, the need to have several of the charged residues in fixed locations can restrict the sequence diversity of the peptides and limit their applications.

**Figure 3:**
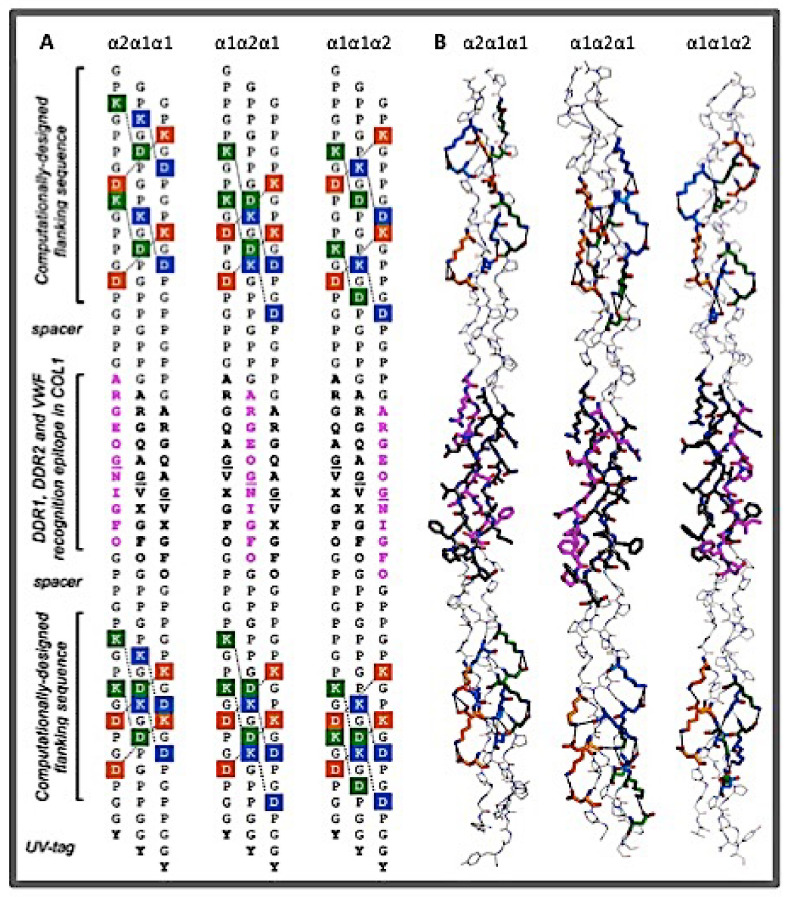
The heterotrimeric peptides modeling the collagen-binding epitopes on type I collagen (21). (**A**) The design and the amino acid sequences of the heterotrimers mimicking type I collagen in three different registers: α2α1α1, α1α2α1, and α1α1α2; here X is norleucine, a methionine bioisostere. The N- and C-termini flanking sequences are generated by a computer program utilizing genetic algorithm to optimize the location of the Lys-Asp charge-pairings in the helix. (**B**) The corresponding crystal structure of the heterotrimers in three registers.

In a new development, it was found that short stretches of peptides of five (Gly-X-Y) tripeptide units consisting of the charge-pairing residues in the optimal positions can be used as a hetero-nucleation site to develop peptides carrying sequences from natural collagens [21]. A set of heterotrimeric peptides in the AAB composition modeling the cell adhesion epitopes or the vWF binding site of type I collagen in all three possible chain alignments were developed. These heterotrimeric peptides have the hetero-nucleation sites at both the N- and the C-termini, flanking 12 residue-specific CBP (collagen-binding protein)-binding epitopes in the center (Figure 3). The specific amino acid sequences of the nucleation sites were computationally generated using a genetic algorithm; a group of Lys and Asp residues were strategically positioned to form a unique set of optimally oriented Lys-Asp salt bridges which stabilize the triple helix having the desired chain alignment. The folded structure was further “covalently captured” by converting the salt bridges into an amide bond between the ammonium group of the Lys side chain and the carboxylate group of the Asp. Based on the structural, stability, and binding studies, the triple helix in the α1α1α2 alignment was found to be less thermally stable than those in the other two possible chain registers, but had the highest binding affinity to DDR1 and vWF. The α1α1α2 heterotrimer was also found to induce higher levels of cellular DDRI and DDR2 kinase activation. These findings appeared to indicate that the α1α1α2, rather than the α1α2α1 as proposed by previous studies, was most likely to be the correct chain alignment of type I collagen. In this conclusion, it is implied that the correct chain register of collagen was not necessarily the one with the highest stability, but the one with highest bioactivity.

The covalently captured heterotrimeric triple helix can extend the ToolKit approach to study the binding and MMP specificity of heterotrimeric collagens. The covalent-capture can potentially be a more effective alternative than Cys-based cross-linking strategies to generate cross-linked heterotrimeric peptides that are more homogeneous, and more stable. Such systems can find a wide range of applications for collagen research.

### 2.3. Applications of CMPs

Collagen rarely functions as an individual triple helix in tissues. To truly “mimic” collagen, CMPs need to further associate into fibril-like supramolecular structures. Developing higher order molecular assemblies through the self-association of CMPs, however, has proven to be challenging: Small sizes and limited sequence diversity are two likely limiting factors.

#### 2.3.1. Self-Assembled Fibrillar Structures

**Figure 4:**
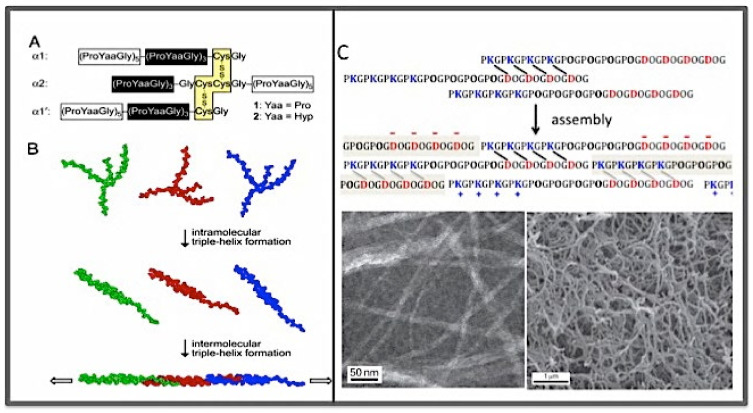
The "sticky end” approach for the self-assembly of collagen mimetic peptides (CMPs). (**A**) The cross-linked CMP with sticky ends. (**B**) The self-assembly process to form a super triple helix [124]. (**C**) The charge-pair directed self-assembly of the KOD peptide, and the TEM images of the KOD fibrils (left, the scale bar is 50 nm), and the hydrogel formed by KOD (right, the scale bar is 1 μm). The directional, interchain Lys-Asp salt brides of the nucleation site are highlighted by short, slanting bars.

A common approach to form a long triple helix through self-assembly is to use the so called “sticky-end” strategy. In the approach by the Raines’ group, the sticky-end CMPs were created using a cross-linked “core” that brought together polypeptide chains with different lengths extending from the C- and N-termini of the core as branches (Figure 4A,B) [124]. The branches were peptides with (POG)_n_ or (PPG)_n_ sequences that had a high propensity to trimerize to form a triple helix. The three strands at the N-terminal end of the core formed an intramolecular triple helix but with an overhang that was complementary to the segment sticking out from the C-terminal end of the core, thus the “sticky ends”. The intermolecular assembly between the complementary sticky ends then produced helices that could reach a length of 200 nm or longer. The functionality of these long triple helices has yet to be fully evaluated. In another approach using disulfide bond cross-links, Koide and colleagues developed long triple helix assemblies containing the integrin-binding epitope GFOGER sequence [125,126]. This supramolecular CMP formed a hydrogel and was found to support integrin-mediated cell adhesion in a fashion “comparable to that of native collagen”.

The sticky end approach used by the Hartgerink’s lab was based entirely on self-association (Figure 4C) [123]. The first successful case was the KOD super-triple helix. The building block was the KOD peptide which had a modular amino acid sequence composition made of three units: (P**K**G)_4_ (the K), (P**O**G)_4_ (the O), and (**D**OG)_4_ (the D). The core of the sticky end was stabilized by a set of Lys-Asp salt bridges. In addition to growing long, the unsatisfied Lys and Asp residues also provided additional interacting surfaces for further lateral association of the triple helices. The result was a hydrogel sharing many features with those created using natural collagens. Furthermore, because of the (GPO)_4_ units in the long helix, the KOD hydrogel was found to be able to activate platelet and clot whole blood plasma [69]. It is one of the CMP materials with a high potential for biomedical applications as a synthetic hemostat. By rearranging the modular units of K, O, and D in a CMP, it was found that the size of the core (the nucleation site) was a crucial design factor that determined if the self-assembly of the CMP would produce hydrogels or amorphous aggregates [127].

A recent work in the Raines’ group further pointed out that the Lys-Asp charge-pair-based sticky-end approach could be optimized by including the “elements of symmetry” in order to afford identical interactions for every peptide in the assembly [122]. Under this symmetry condition, all Lys and Asp residues engage in interchain charge–pairing interactions, thus maximally stabilizing the assembly and at the same time eliminating unpaired Lys and Asp residues that are prone to form non-specific aggregates. The symmetry consideration was developed into a set of design rules for CMPs that could self-assemble into a long triple helix. As it was shown in their study, peptides generated using these design rules formed long triple helices that could match or even exceed the natural collagen in length.

It worth pointing out that the self-assembled long triple helices or fibrillar structures of CMPs have one fundamental difference from that of the collagen fibrils. Collagen fibrils are assembled through lateral, staggered association of triple helices. While these self-assembled triple helices are wonderfully long, they lack certain structural elements of the native collagen fibrils, chief among them the axial structure of the *D*-period. Similarly, the mechanical support provided by the CMP hydrogels are likely to be different from the support of the collagen molecular scaffold in the ECM, despite similar morphology as shown by TEM images. None of the supramolecular CMPs have the *D*-period-like structure seen in collagen fibrils. In one report, it was proposed that a *D*-periodic microfibril was formed from blunt-end self-assembly of a 36-resdiue triple helix [128]. However, the proposed 17.9 nm *D*-period of the microfibrils is nearly twice the length of the constituent triple helix. The microfibrils must have formed through a very different molecular arrangement than the *D*-periodic collagen fibrils.

#### 2.3.2. Interaction of CMPs with Damaged Collagens

There have been many interesting studies using CMPs to produce nano-structures in different shapes, sizes, and compositions. Some of these molecular assemblies have shown promise for biomedical and other applications [129,130,131,132,133,134,135,136,137,138,139]. There is a good account of some of these works in a recent review [56]. We would, however, like to end this section on synthetic CMPs with the application of CMPs that covers the other end of the spectrum—by going small. This application uses CMPs in an unfolded, single chain conformation to study damaged collagen in tissues.

As indicated in some of the studies above, multiple (Gly-Pro-Hyp) peptide repeats have a high tendency to trimerize with two other peptides to form a triple helix. This tendency makes it a high affinity ligand which can bind to unfolded or partially unfolded collagens. Based on this property, Yu and colleagues have developed several effective peptide probes to detect damaged collagen using a (POG)_9_ peptide with a fluorophore conjugated to the N-terminus [140,141]. These collagen hybridizing peptides (CHPs) were found to have a high tendency to form a hybridized triple helix with unfolded chains in damaged collagen, both under in vitro and in vivo conditions. The CHPs are also remarkably stable and resistant to proteolysis in serum. However, the high propensity of (GPO)_9_ for triple helix formation has its downside in this application, since the affinity to damaged collagen diminishes once the CHPs trimerize themselves to form a triple helix. To prevent the self-trimerization, the peptides had to be heated above 80 °C before injection for in vivo applications; the *T_m_* of a (GPO)_9_-based triple helix can reach above 50 °C. To overcome this problem, new features were included in the CHPs. In one clever approach, a nitrobenzyl (NB) group was attached to the backbone nitrogen of a Gly residue located at the center of the peptides [142]. This “NB-caged” CHP cannot trimerize to form a triple helix due to steric clashes of the bulky NB group. Once delivered to the site of detection, the NB group can be removed by a brief radiation of UV light and free the CHPs so they can hybridize with damaged collagen. In a more recent approach, 2*S*,4*S*-fluoroproline (flp, or f) was used to replace Pro [143,144]. The homotrimer (GfO)_9_ has a low stability at room temperature, but its strands can effectively bind damaged collagen [145]. The range of applications of the CHP probe is remarkable. It has been used to visualize matrix turnover caused by proteolytic migration of cancer cells in a 3D collagen gel, and to detect the ECM changes associated with mechanical stress, different types of cancers, and connective tissue diseases in mouse models [143,146,147].

Works in the Raines’ lab utilized a similar concept to develop CHP probes that can hybridize to the cell surface collagenous protein of *Streptococcus pyogenes*, which is a bacterium responsible for serious infections of wounds and connective tissues [148]. These CHPs utilized (GPP)_7_ peptides, which have a low tendency for trimerization (thermal stability of (GPP)_7_ triple helix < 27 °C), but maintain the ability to hybridize with damaged collagen at room temperature. Such CHPs can potentially be used to detect bacterial infection at the wound bed. Another modified CHP with the sequence Cy5-G(SG)_2_-(fOG)_7_ was used to assess the wound healing and tissue remodeling process in injured human skin, the burned damages in tissues, and the abnormal ECM in bone associated with developmental detects [149,150,151,152].

## 3. The Recombinant Collagen Peptides

There has been an increase in the use of recombinant peptides produced by a recombinant system using designed genes for collagen research. The CMPs produced by chemical synthesis are often limited to <50 residues; the need to include multiple GPO or GPP sequences for stability and folding further limited their ability to model natural collagens, which frequently have more than 1000 amino acid residues per a single polypeptide chain. Because of the feasibility of including large stretches of amino acid sequences to model more extended ranges of collagen, researchers have turned to the recombinant peptide to gain, almost literally, a broader view of collagen and of the mechanisms of its functions. The expression system of *Escherichia coli* (*E. coli*) is often the system of choice. The simpler prokaryotic genome is easier to manipulate and to achieve a high yield [153]. The obvious limitation is the lack of post-translational modifications, and for collagen, a lack of Hyp in particular. However, emerging studies have showed that collagen mimetic peptides without Hyp can still interact with integrin, support cell adhesion, be recognized by MMP and be cleaved on the same site, and self-assemble into fibrils [96,154,155,156,157]. Another limitation is the ability to generate heterotrimers, although exciting new progress has been made in this account (details are given in the next section). In general, the bacterial expression system of collagen peptides is considered a valuable tool for collagen research, albeit not perfect.

We would like to clarify that the recombinant peptides are not recombinant collagens. There have been many works in the recent decades devoted to producing full-chain human collagen molecules using an expression system. In such cases, an entire gene(s) of a human collagen (or of another organism) was cloned into a host expression system. The collagen molecules so produced were meant to be an exact replicate of human collagen. Such recombinant collagens have great potential for many applications; a full chapter in this special issue is devoted to such recombinant collagens. Here, we focus on collagen-like peptides with designed amino acid sequences ranging from ~30–300 amino acid residues in size, which can be produced either by a eukaryotic expression system or, more frequently, by a prokaryotic expression system.

### 3.1. The Sequence–Stability Relationship Revisited

Further expanding their work on the triple helix propensity of amino acid sequences, Brodsky and coworkers analyzed the different factors in the overall thermal stability of a series of recombinant peptides derived from bacterial collagen Scl2 [155,158,159,160]. The size of the triple helix domain in the peptides ranged from 75 to 237 residues. Despite lacking any hydroxyproline, these recombinant collagens were stable, with a *T_m_* between 23.5 and 35.6 °C depending on the specific sequence and the length of the peptide. The unexpected stability was attributed to the high content of Pro in X positions, and the high level of charge-based interactions. As a result, the *T_m_* was found to be sensitive to pH and the ionic strength of the buffer; a 3–14 °C decrease was reported for some peptides as the pH was reduced from 7 to 2.8 [158]. The stability was found to increase with chain length and reach a plateau when the size reached about 150 residues. For peptides longer than 150 residues, the value of the *T_m_* was more or less stabilized at 36–39 °C with less dependence on the amino acid sequence. Replacing the stabilizing Pro residues with residues with a lower propensity reduced the thermal stability of the triple helix, although the exact extent of change in the *T_m_* did not quantitatively follow the simple additive rules of short CMPs [155].

#### 3.1.1. Defining the Sequence Requirements of Fibronectin Binding, and of the Proteolysis of MMPs

The ability to include longer stretches of a native sequence of collagen makes it possible to study interactions of collagen with larger molecular complexes using the recombinant peptides. Peptides derived from Scl2 are particularly good as a model for such studies. Because of its prokaryotic origin, Scl2 has a low affinity to the human collagen receptors and low susceptibility to MMPs. For this reason, Scl2 was often considered a collagen “blank slate” [160].

By including an amino acid sequence of 3 to 8 Gly-X-Y triplets (9–24 residues) taken from type II collagen in the center *guest* site of Scl2, an 18-residue amino acid sequence was identified as the binding site of fibronectin on type II collagen [161]. Fibronectin is a large, dimeric glycoprotein that interacts with collagen to maintain the integrity of the ECM. The 18-residue binding site of fibronectin is nearly three times the size of the footprints of the collagen-binding domain of integrin or of vWF on collagen, and would be very difficult to characterize using short CMPs. Similar studies of the recombinant peptides with other collagen receptors further expanded our understanding of the molecular recognition process of collagen [154,162].

The Scl2-based peptides were also used to define the sequence selectivity of MMPs [163]. Peptides with an insertion of 12–18 amino acid residues from type III collagen at the *guest* site of Scl2 were produced as the substrate of MMP-1 and MMP-13. A 5-tripeptide sequence was identified as the minimum requirement for MMP digestion, including 4 residues before and 11 residues after the cleavage site. The ratio of *k_cat_*/*K_m_* of the reaction was close to that observed using human collagen as a substrate, but the values of both *K_m_* and *k_cat_* were about 10-fold higher. This discrepancy was partially attributed to the lack of Hyp in the recombinant peptides, which can affect the affinity of the peptide as a substrate. The same cleavage site and the high *k_cat_* indicate the mechanism of the enzymatic reaction is similar for the two substrates.

#### 3.1.2. The Impact of Gly Substitution Mutations

CMP 30–45 residues in size have been used to elucidate the structural and stability effects on collagen by mutations linked with brittle bone disease (osteogenesis imperfecta or OI). The majority of OI mutations are missense mutations that lead to the replacement of the obligatory Gly to a different amino acid. One unique feature of the OI mutations is that the same type of Gly substitution often results in phenotypes of the disease with very different severity depending on the location of the Gly. Because of their limited sizes, studies using CMPs could not fully resolve why the location of a mutation has such a profound impact. In one study, a recombinant peptide containing the 63-residue Hyp-free region of the α1 chain of type I collagen was produced using a bacterial expression system [164]. Several Gly substitution mutations in this region were linked to OI with very different phenotypes. For the same Gly to Ser substitution, the OI was mild when it takes place at Gly^901^, but just 12 residues away at Gly^913^ it causes a severe type of OI characterized by prenatal death. In this 90-residue model peptide F877, the authors were able to demonstrate that the structural impact of the Gly replacing mutation is modulated by the local stability and sequence context of the mutation site. Located next to a relatively unstable region consisting of no imino acids, the conformational alteration related to the Ser substitution at Gly^913^ triggered a large scale unfolding of the triple helix, while the effects at Gly^901^ were better confined to the close vicinity of the mutation site. A region of more than 20 residues in size was found to be unfolded because of the substitution of Gly^913^. Conformational change of this scale is difficult to study using short CMPs. In a study using the Scl2 consisting of about 300 residues, Brodsky and coworkers were also able to demonstrate that the relative location of the Gly substitution site to the folding nucleation domain had a different overall impact on the structure and stability of the triple helix [165,166].

### 3.2. The Heterotrimeric Recombinant Peptides

The recombinant peptides offer a different strategy to make heterotrimers by utilizing a heterotrimeric nucleation site appended to the triple helical domain. The nucleation site functions as the C-propeptide of type I or type IV collagen to bring together three different polypeptide chains. Recent studies of the non-collagenous domains of collagen type IX indicate that the NC2 domain of collagen type IX can function as a nucleation site of heterotrimers [167]. Collagen type IX is a heterotrimer consisting of three different chains, and is a member of the fibril-associated collagens with interrupted triple helices (FACIT). The isolated NC2 domain itself consists of 3 different polypeptide chains, each about 38 residues long, and forms a stable α-helix coiled coil. It was further demonstrated that the NC2 domain can be used to direct the folding of a hetero-trimeric triple helix containing the vWF binding site of type I collagen [106,168]. For each peptide chain of the NC2 domain (IXα1, IXα2, and IXα3), two fusion proteins were expressed by connecting to either the peptide mimicking the α1 chain of type I collagen (Iα1) or the one mimicking the α2 chain (Iα2). For instance, by mixing Iα1-IXα1, Iα1-IXα2, and Iα2-IXα3 in a 1:1:1 ratio, a molecular chimera forms with the triple helix domain with a specific register referred to as *112*. It should be noted that, since the chain register of the NC2 domain is not known, the register *112* is not the same as α1α1α2, which generally denotes the chain alignment with the two α1 chains in the leading and middle positions, and the α2 in the trailing. However, by simply mixing the group of 6 different fusion proteins, the vWF domain in all three different chain registers can be produced. After stability and binding studies, the heterotrimer with the *112* arrangement showed the highest thermostability and the highest binding affinity. It was, therefore, concluded that by connecting the peptide Iα1 to the IXα1 and IXα2 chains, and peptide Iα2 to the IXα3 chain, the NC2 domain will lead to the formation of a heterotrimer in the same chain register as that of native collagen type I [167]. This approach to heterotrimers is still in its early stages; further studies are needed to fully establish its effectiveness and applicability.

### 3.3. The Collagen Mimetic Fibrils

**Figure 5:**
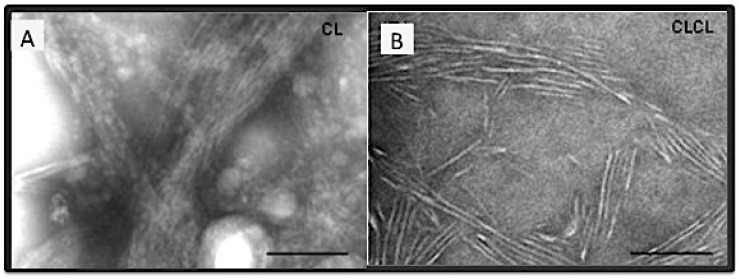
The self-assembled fibrils of CL and CL-CL peptides. (**A**) The mesh-like aggregates of CL. (**B**) The discrete, end-on-end assembly of CL-CL [159]. The scale bars are 100 nm in both pictures.

The longer triple helices with more diverse amino acid side chains on its surface also facilitated the lateral self-association of the triple helix. Using bacterial collagen, Brodsky and coworkers showed that peptides with a 79 GXY tripeptide can form fibrous bundles at neutral pH (Figure 5) [159]. When the size of the peptide was doubled to include two identical triple helix units, in a construct of CL-CL where CL = 79 tripeptides, the peptide forms a more discrete structure characterized by an in register, end-on-end self-assembly (Figure 5). The length of the end-on-end assembly is ~140 nm, which is in a good agreement with that of a CL-CL molecule; the diameter of the assemblies is 4–5 nm which is about 4× that of a single triple helix. It is not clear if the triple helices are in a parallel arrangement as in fibrillar collagen. This work demonstrated that triple helices in the range of ~100 residues in size can self-associate to form stable fibril structures even in the absence of Hyp. Previous work seemed to indicate that interactions involving the hydroxyl of Hyp are necessary for the stabilization of the fibril assembly [131].

**Figure 6:**
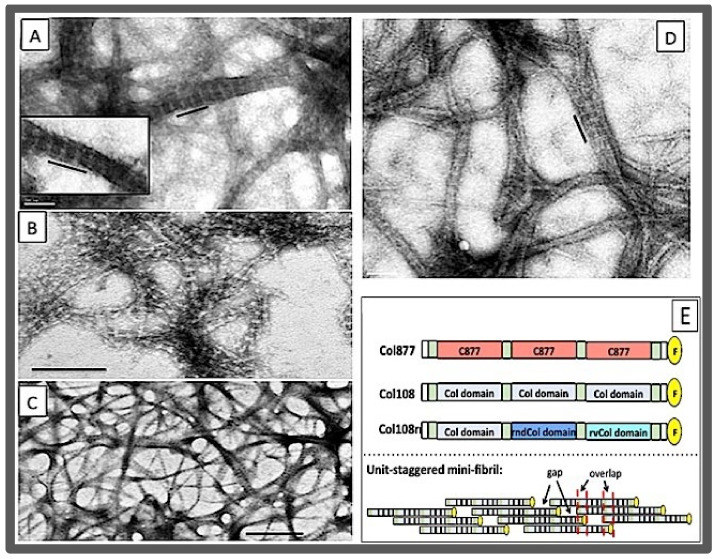
The unit-staggered mini-fibrils with a 35-nm *d*-period. (**A**–**D**) TEM images of Col108 mini-fibrils (13). The scale bars are 100 nm in (**A**,**D**), 200 nm in (**B**), and 500 nm in (**C**). (**E**) The schematic depictions of the peptides and the unit-staggering of the mini-fibril. The identical sequence units (SUs) are shown as rectangles in the same color: Col877 (3 red), Col108 (3 light blue), Col108rr (3 different SUs in three different colors); the yellow circles represent the foldon domain; the (GPP)_4_ sequence that is present in the N-terminus of each SU is shown in green blocks (13). The unit-staggered mini-fibrils are drawn to highlight the alternating gaps and overlap zones, and the in-register alignment of the interacting residues (black vertical bands).

The self-assembly of the axial repeating structure of collagen fibrils indicates the specific *D*-staggering arrangement representing the most stable conformation during the self-association of the triple helix. Thus, in addition to providing sufficient stability for mutual association, there ought to be a built-in mechanism for conformational bias for the specific staggered structure. This bias was achieved by using recombinant peptides with repeating sequence units (SUs) in its primary structure [13,169]; each SU consists of 123 amino acid residues in an uninterrupted (Gly-X-Y)_n_ repeating sequence. Two collagen mimetic peptides: Col108 and 2U108 with, respectively, 3 and 2 identical SUs arranged in tandem plus a C-terminal overhang region were shown to self-assemble into mini-fibrils with an axial repeating structure of 35 nm as examined using electron microscopy (Figure 6A–D) and AFM [13]. This 35-nm repeating structure of the mini-fibrils consists of a ~15-nm overlap region and 20-nm gap, and is designated the *d*-period to distinguish it from the 64 nm *D*-period of natural fibrillar collagen. These mini-fibrils can reach 1 μm in length with a diameter about 50–70 nm. In a sense it is a self-assembly at an entirely different scale compared to the self-assembled long triple helix of CMPs. Structural analysis and biophysical studies have further indicated that the 35 nm *d*-period results from the self-assembly of the triple helices with a mutual staggering of 1 SU at the ends, in a way reminiscent to that of the *D*-staggering of collagen fibrils [13].

The design of the Col108 and 2U108 was based on the idea that, in the linear conformation of the triple helix, the structural periodicity of the fibrils should come directly from sequence periodicity. The tandem placement of the identical SUs, each consisting of 123 residues, determines a 123-residue sequence periodicity in the primary structure of Col108 and 2U108: Residues in the first sequence unit are repeated three and two times, respectively, every 123 residues. Several lines of observations support the sequence periodicity based *d*-period of the mini-fibrils:

(1) The size of the *d*-period was in a good agreement with the size of the section of triple helix formed by one SU (123 amino acid residues, or 41 GXY triplets) based on the average helical rise of 0.86 nm per GXY tripeptide. Furthermore, the overlap region was in good agreement with the size of the C-terminal overhang;
(2) Another peptide, the 1U108, contained only one SU. This peptide did not have a 123-residue sequence periodicity and did not form fibrils [169];
(3) The pattern of sequence periodicity was the determining factor. In another peptide, peptide Col108rr, the sequences in each of the three SUs of Col108 were shuffled such that while the amino acid composition of this peptide was the same as Col108, the sequence periodicity was lost. As expected, the Col108rr only formed non-specific aggregates [170];
(4) In a new peptide, peptide Col877, the SU of Col108 was replaced by another 123 residues containing residues 877–986 of the α1 chain of human type I collagen [170] Thus, in contrast to Col108rr, Col877 had a very different amino acid composition from that of Col108, but had the same 123-residue sequence periodicity in its primary structure. Remarkably, Col877 can form mini-fibrils with the same 35-nm *d*-period as Col108. The Col877 mini-fibrils are a clear demonstration that the unit-staggering arrangement is at the foundation of the *d*-period axial structure.
(5) The Col877 mini-fibril, and the lack of the fibril assembly of Col108rr and 1U108 indicated the foldon domain was not the determining factor of the fibril assembly, since all peptides, including Col108 and 2U108, contained the foldon domain.

The important finding of the unit-staggered mechanism of the mini-fibrils is that the *D*-staggered fibril assembly of collagen depends on the properties of the triple helix per se. Although factors such as the Hyp and telopeptides can facilitate the fibrillogenesis process, they may not be the determining factors [47,156,157]. The large stretch of sequence of natural collagen in Col877 also means the Col877 mini-fibrils can potentially be a model system to study binding and the effects of OI mutations on type I collagen at the fibril level.

## 4. Conclusions

The knowledge of the structure, and the sequence–function relationships of the triple helix have led to a wide range of applications for CMPs. One particularly productive area of research is the use of CMPs to probe cell–ECM interactions. The next immediate challenge is to understand and to replicate the modulation of these interactions by the supramolecular structure of collagen in the ECM. For this purpose, a model of a collagen fibril is needed to investigate (1) the relationship between the supramolecular structures of collagen and the biomechanical properties of the ECM, and (2) the determining factors of the molecular assemblies of collagen. Fibrillar collagen has been the focus of the effort to understand the molecular properties of the ECM because of its abundance, and because collagen fibrillogenesis is largely a self-association process of the triple helix without the involvement of any other globular domains. To fully capture the mechanical properties and the biological activity of collagen fibrils, the CMP-based systems most likely need to have a *D*-period like axial structure.

The research of CMPs is often inspired by the goal of developing synthetic collagen, or synthetic ECM. In addition to their far-reaching impact on biomedical applications and on applications in bioengineering, the ability to create complex systems like collagen stands as the ultimate test of our understanding of protein, of protein design, and of chemical synthesis. These are complicated systems. Collagen fibrils, for example, are heterotypical assemblies in tissues consisting of many different kinds of collagen and other macromolecules. The achievements we have gained so far in creating fibril-forming CMPs, or even creating a *D*-like structure by design, are rather modest; they are exceedingly simplified structures compared to natural collagen. But it is often such simplified model systems that aid us in peeling away the complexity of the tissues and the ECM one layer at a time. The allure of a collagen mimetic, after all, is functionality without the complexity.

## Data Availability

Not applicable.
